# Examining the Popularity, Content, and Intersections With the Substance Abuse and Mental Health Services Administration’s Definition of Recovery in a Nonclinical Online Cannabis Cessation Community: Infodemiology Study of Reddit Posts

**DOI:** 10.2196/47357

**Published:** 2024-09-27

**Authors:** Elyse J Thulin, Maureen A Walton, Erin E Bonar, Anne Fernandez

**Affiliations:** 1 University of Michigan Ann Arbor, MI United States

**Keywords:** cannabis use disorder, online community, cannabis, human-computer interaction, mobile phone

## Abstract

**Background:**

Cannabis consumption has increased in recent years, as has cannabis use disorder. While researchers have explored public online community discussions of active cannabis use, less is known about the popularity and content of publicly available online communities intended to support cannabis cessation.

**Objective:**

This study aims to examine the level of engagement and dominant content of an online community for cannabis cessation through 3 specific aims. First, we examine the use of a subreddit cannabis cessation community (r/leaves) over time to evaluate the popularity of this type of resource for individuals who want to stop using cannabis. Second, we examine the content of posts in the community to identify popular topics related to cessation. Third, we compare the thematic findings relative to the 4 domains of recovery defined by the Substance Abuse and Mental Health Services Administration (SAMHSA). By examining these 3 gaps, we take the initial steps toward understanding the experiences being shared online among individuals interested in cannabis cessation and compare them with the principles outlined in the SAMHSA definition of recovery.

**Methods:**

Using the Pushshift application programming interface, we collected the count of posts by year between 2011 and 2021 and the narrative of the 100 posts with the most comments per year in a popular cannabis cessation–focused subreddit (r/leaves). A linear model and a nonlinear model were compared to evaluate change in the number of posts by year. Mixed natural language processing and qualitative analyses were applied to identify top terms, phrases, and themes present in posts over time. Overlap between themes and the 4 SAMHSA domains of recovery (health, purpose, community, and home) were examined.

**Results:**

The number of annual posts in r/leaves increased from 420 in 2011 to 34,841 in 2021 (83-fold increase), with exponential growth since 2018. The term that was the most common across posts was “smoke” (2019 posts). Five major themes were identified, and a narrative arc was represented, from motivations and perceived benefits of cannabis use to the negative consequences of use, strategies to change behaviors, and the positive and negative consequences of change. There was substantial overlap between these 5 themes and 3 of SAMHSA’s 4 domains of recovery: health, purpose, and community. However, the domain of home was less commonly identified.

**Conclusions:**

Engagement in this online cannabis support community appears to be increasing. Individuals using this forum discussed several topics, including multiple aspects of recovery defined by the SAMHSA. Online communities, such as this one may, serve as an important pathway for individuals seeking to reduce or cease their consumption of cannabis.

## Introduction

### Background

The prevalence of cannabis consumption and clinical diagnoses of cannabis use disorder (CUD) has increased in the past 2 decades [1-3]. The intensification in delta-9-tetrahydrocannabinol potency of cannabis products in recent years, expanding types of consumption modalities of high-potency products (eg, vaping, dabs, and edibles), and increased perceived acceptability of cannabis use increase the risk for greater use and dependence on cannabis [4-12]. CUD has serious consequences for individuals, families, and communities, including increasing the number of disability-adjusted life years, indicating lower quality of life [13]. However, compared with other substance use disorders, cannabis use change (eg, cessation and reduction) is under-studied [14].

As noted by the Substance Abuse and Mental Health Services Administration (SAMHSA), recovery is often more than changing one’s substance use behaviors [15]. Rather, recovery is a multidimensional process related to health (making choices that support physical health and psychological well-being), community (having relationships that yield support, care, and hope), purpose (engagement with meaningful daily activities, such as work, school, or other participation in society), and home (access to a stable, safe space to live). Within SAMHSA’s definition, recovery is achieved through self-directed changes that enable individuals to navigate their lives, improve their health and well-being, and work toward their full potential. While there are a handful of evidence-based clinical psychotherapies for CUD [16-18], approximately 85% of individuals who would qualify as having a diagnosis of CUD never receive clinical treatment [3]. Cost, geographic access, perceived social stigma, internalized stigma, and a lack of knowledge of or low belief in the efficacy of treatments are all barriers to treatment [19,20]. Given the increase in cannabis use and low rates of clinical treatment, we aim to investigate cannabis use behavior changes outside of the clinical context and how these behavior changes may or may not intersect with the domains of SAMHSA’s multidimensional definition of recovery.

### Multiple Pathways to Recovery

Traditionally, the focus of substance use behavior change within clinical and nonclinical settings (eg, mutual help organizations such as Alcoholics Anonymous) has been the prolonged cessation of all substances [21]. While long-term cessation is beneficial for a proportion of the population who desires to change their substance use, focusing on the singular outcome of cessation of all substances has created barriers for individuals to engage with alternative behavior change pathways (eg, medically assisted treatment and behavioral harm reduction strategies) [22]. To reduce barriers to recovery, in 2013, the SAMHSA expanded its definition of recovery to include multiple pathways and desired outcomes of substance use change and identified the holistic nature of change as it relates to the 4 domains of health, purpose, community, and home [23,24]. Identifying the intersection of cannabis change in nonclinical contexts and the 4 domains of SAMHSA’s definition of recovery may yield facilitators and barriers to nonclinical routes of cannabis use behavior change, which could then be used in future health interventions to expand access and relevance of programs within and outside of clinical contexts.

### Online Spaces Promoting Cannabis Cessation

There is evidence that those wanting to reduce or cease cannabis use are increasingly turning to publicly available online support forums [25,26]. Unique to online forums is the opportunity for interactions to occur organically and asynchronously within an individual’s daily life [25]. Studying online communities related to cannabis use behavior change is relevant for 3 overarching reasons. First, unlike in-person forms of peer support (such as mutual help organizations) or social support provided through formal treatment programs, online communities are not limited by geography. This online access is a particularly salient opportunity for individuals living in rural or underresourced settings who may benefit from online care and support [27]. Second, some online communities provide the opportunity to participate and receive support without disclosing one’s personal identifying information, including one’s physical appearance. Maintaining pseudonymity may be appealing to individuals who desire social support and advice while simultaneously maintaining anonymity because of the perceived stigma associated with substance use disorders [22]. Finally, online spaces are already in existence, are being used worldwide, and are becoming ever more popular over time [28]. Understanding the content and utility of these spaces could guide future research and inform clinical recommendations for new interventions or additional support [29].

The few studies that have examined online cannabis communities have focused on posts from individuals who are actively using cannabis (as opposed to focusing on cessation or reduction) [30-32]. These studies found that Reddit contains multiple procannabis use communities (called subreddits), which have documented increases in cannabis references across time and discussions about newer forms of delivery (eg, vaping and dabbing). In contrast, one study investigated the desire to cease cannabis use in a subreddit community focused on recovery (r/leaves) [33]. In this study, 38% of individuals who posted to this forum reported symptoms aligned with the *Diagnostic and Statistical Manual of Mental Disorders, 5th Edition* classification of mild CUD severity, 16% with moderate severity, and 12% with severe severity. Though setting the foundation that this community is composed of individuals who are experiencing dependency on cannabis and want to change, research is needed to investigate the relevancy and value of this online community to those interested in recovery and to provide information on recovery from the perspective of those with lived experience. Such data could expand our understanding of how these communities may be of use to individuals with CUD, which could inform future integration or creation of online support groups as treatment adjuncts.

### Objectives

Given the negative consequences associated with CUD, examining the use of such nontraditional self-help–based online forums is important to expand understanding of potential recovery pathways. In this study, we aim to address 3 gaps in the research. First, we examine the use of a subreddit cannabis cessation community (r/leaves) over time to evaluate the popularity of this type of resource for individuals who want to stop using cannabis. Second, we examine the content of posts in the community to identify popular topics related to cessation. Third, we compare the thematic findings relative to the 4 domains of SAMHSA’s definition of recovery. By examining these 3 gaps, we take the initial steps toward understanding the experiences being shared on these sites among individuals interested in cannabis cessation and compare them with the principles outlined in the SAMHSA definition of recovery.

## Methods

### Data Source

Reddit is an online platform that individuals use to gather information and connect with others [34]. Reddit is composed of many community forums (called subreddits), and the platform as a whole has consistently increased in popularity over the past decade [35,36]. Many subreddit communities allow for posts and comments to be viewed without an account (ie, allowing for general exploration). Finally, individuals who create accounts generally use pseudonyms on their profile, thus avoiding disclosure of personal identifying information (eg, name, location, and physical appearance) [37].

We extracted data from Reddit using the Pushshift Reddit application programming interface (API) from the r/leaves subreddit community [38]. Using this API, we collected the number of posts by year and the text of the 100 posts with the greatest number of comments per year. The r/leaves subreddit thread is described as “a support community to help stop smoking cannabis, marijuana, pot, weed, edibles, or getting high.” Though many communities reference cannabis-related terms (eg, cannabis, marijuana, and weed) and multiple communities that are dedicated to cannabis-related topics, r/leaves appears to be the most frequently used community dedicated to cessation of cannabis use [30-33]. This community was started on January 8, 2011, and as of June 2022, it had approximately 240,000 members. Though demographic statistics for this subreddit are not available, from our initial review, we found that all posts are written in English. Broadly, the known statistics of Reddit users indicate that over half of them are from the United States, and the distribution of users skews younger and male [39].

### Data Extraction

First, we gathered the top 100 most–commented-on posts for each year within the r/leaves community. We only collected original posts, as opposed to comments, which have their own set of popularity rankings. Given prior research findings that a small number of community members post with high frequency (and thus, posts may not be representative of a wider set of community members) [40], we determined the number of unique users who authored the top posts. We noted an archival issue in Pushshift for the year 2013, which prevented the collection of posts from that year; thus, our data includes the top 100 most–commented-on posts by year for 2011, 2012, and 2014 to 2021 for 1000 posts.

### Data Analysis: Top Terms and Phrases

To accomplish the first stated goal (understanding the change in use of this forum across time), we calculated the annual number of original posts by year from 2011 to 2021. We plotted the number of posts by year to visualize the trend of posts across time, compared coefficient significance, and adjusted *R*^2^ values in linear versus quadratic models to assess which had the better fit.

Our second goal was to identify the top themes in the most popular posts (those with the greatest number of comments). To do so, we used mixed computational and qualitative methods [41,42]. Given the importance of input from individuals with lived experience with substance use disorders and recovery, the lack of research on cannabis recovery online forums, and the frequent use of clinical samples in the general field of recovery, we chose to examine the most frequent terms and used an inductive qualitative coding approach to allow the data to guide our findings. Once we had the emergent themes present in this online community, we then examined the intersection of these themes with existing SAMHSA recovery definition domains (health, purpose, community, and home). First, we preprocessed text (ie, case standardization, removing punctuation and numbers, and using the stems of all words) using standard procedures [43]. We removed the most common English words that do not have significant meaning (eg, the and a) using the System for the Mechanical Analysis and Retrieval of Text stopword dictionary [44] through the R “quanteda” package [45]; for phrases, stop words were not removed to increase accuracy and interpretability of multiword findings [46]. Preprocessing of text increases the accuracy and parity of natural language processing techniques and increases the replicability of findings [43]. Data scraping and analyses were conducted in R (version 4.1) [47].

### Data Analysis: Emergent Themes in Posts

After identifying the top terms and phrases present in preprocessed posts, we randomly selected 10% (100/965) of posts to review to help contextualize findings; mixing natural language processing and qualitative methods increases the accuracy of findings and the ability to analyze a greater amount of text [42]. In line with thematic content analysis [48], the lead author read the subset of posts, created a codebook from the data, and then coded the data using the established codebook; the results from this coding process were reviewed by coauthors (2 of whom are licensed clinical psychologists and 1 of whom is a community psychologist) for saliency and relevancy. Finally, we compared the emergent themes with the 4 domains (health, purpose, community, and home) of SAMHSA’s definition of recovery.

### Ethical Considerations

This study was determined as unregulated by the institutional review board at the University of Michigan. As data are publicly available and we did not have study participants, informed consent was not collected and no compensation was made. Though the data contain usernames (which are frequently pseudonyms), to uphold privacy we do not report any of those usernames in our work. To uphold deidentification and follow Reddit’s intention of anonymity, the quotes presented below are paraphrased based on the reviewed data [49]. Finally, generative artificial intelligence was not used in any aspect of this study (data scraping, cleaning, manuscript drafting, etc).

## Results

### Overview

**Figure 1:**
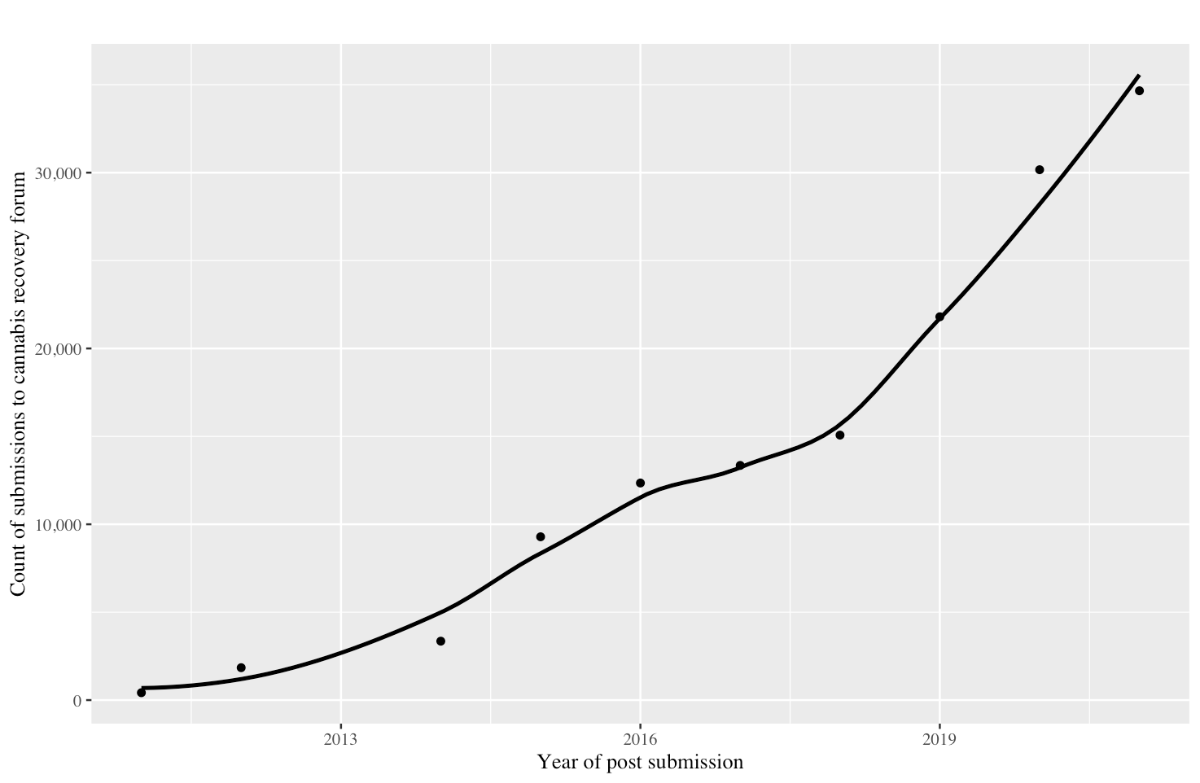
Descriptive count of posts from the Reddit cannabis recovery forum, covering the period from 2011 to 2021.

The number of posts in r/leaves by year increased by 8195% from 420 in 2011 to 34,841 in 2021. In the linear model, for every increase in year, the number of posts increased by 3379.3 (SE 341.3; *P*<.001; Figure 1). The linear model accounted for 91.5% of the variance. In the quadratic model, the linear (β=–1,179,000.0, SE 230,900.0; *P*=.001) and squared coefficients (β=293.1, SE 57.27; *P*=.001) were both significant. The interpretation of these values suggests that the starting point is low (eg, intercept) and increases as the curve opens upward (signified by the positive valence of the quadratic term). The quadratic model accounted for 98% of the variance.

### Top Terms and Phrases

**Figure 2:**
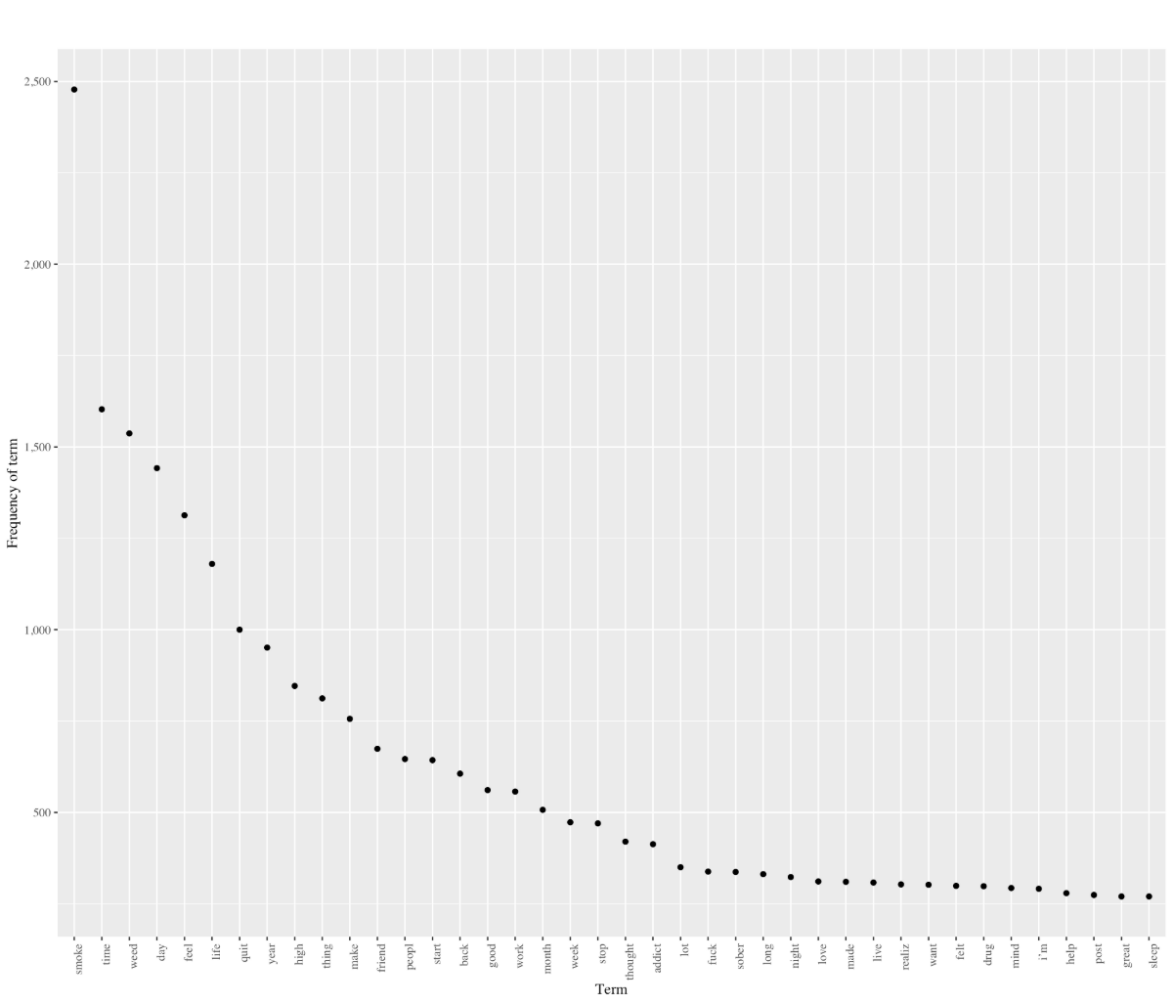
Descriptive frequency of the most common terms in the top 100 most-liked posts from the Reddit cannabis recovery forum posted from 2011 to 2021.

Overall, 1000 posts were collected, representing the 100 most–commented-on posts per year across 10 years (excluding 2013, as noted in the Data Extraction section); 965 of the posts included text (as opposed to other media, such as a video link or only a title) and were analyzed in this study. After text preprocessing, there were 7829 unique features (eg, terms). The top 5 terms across all posts were “smoke” (n=2763), “time” (n=1756), “weed” (n=1738), “day” (n=1635), and “feel” (n=1456). A review of terms produced several clustering of topics related to time (eg, day, month, and year), cannabis use (eg, smoke, weed, and high) and perceived identity related to cannabis use (eg, addict and sober), cannabis behavior change (eg, quit, begin, start, and stop), cognitive functioning (eg, thought and sleep) or emotions (eg, happy and hope), mental health (eg, depress and anxieti, which is the root for permutations of “anxiety”), social purpose (eg, job, school, and money), and terms relationships (eg, friend and famili). The top 40 terms are shown in Figure 2.

Phrases were defined as strings of 3 to 6 words**,** given the limited interpretability of 2-word strings; notably, stemming at times results in the atypical presentation of words (eg, “decid,” the stem of “decided,” “deciding,” and “decide”). The top phrases were “i want to” (n=287), “a lot of” (n=173), “i need to” (n=140), “when i was” (n=118), “be abl to” (n=117), and “i use to” (n=114). The statements expressing want often referred to outcomes of cessation that the individual hoped to experience, including “I want to feel happy,” “I want to be able to remember my time,” and “I want to find a new job.” Many of the top phrases were I statements, such as “i feel like” (n=93), “i have been” (n=82), “i had a” (n=81), “i had to” (n=78), “i don’t want” (n=71), “i was a” (n=65), “i decid to” (n=63), “i just want” (n=58), and “i don’t know” (n=54). Several of the phrases referenced cannabis use behaviors, such as craving (eg, “want to smoke”; n=67) and desire to cease (eg, “to stop smoke”; n=54). There are also multiple references to behaviors related to cannabis cessation, including “tri to quit” (n=64) and “want to quit” (n=61). The top 50 phrases are presented in Table 1.

**Table 1:** Top 50 two-to-three-word phrases in cannabis cessation online community posts (N=1000)^a^.

Phrases used in posts	Phrase across posts, n
“i want to”	287
“a lot of”	173
“i need to”	140
“when i was”	118
“be abl to”	117
“i use to”	114
“want to be”	113
“in my life”	111
“all the time”	102
“of my life”	99
“i feel like”	93
“just want to”	91
“don’t want to”	82
“i have been”	82
“i had a”	81
“i had to”	78
“the first time”	77
“you want to”	76
“i don’t want”	71
“to get high”	68
“in the morn”	68
“i have a”	67
“want to smoke”	67
“i was a”	65
“for the first”	65
“my life i”	64
“that i was”	64
“the end of”	64
“tri to quit”	64
“i decid to”	63
“you need to”	62
“want to quit”	61
“one of the”	60
“hang out with”	60
“to be a”	59
“it was a”	59
“i just want”	58
“feel like i”	58
“a long time”	57
“want to do”	56
“i just want to”	56
“i don’t want to”	55
“in the past”	54
“becaus i was”	54
“i don’t know”	54
“to stop smoke”	54
“for a while”	53
“this is a”	53
“you have to”	53
“i was in”	52

^a^Given the sparsity of term and phrase matrices, providing a percentage of the count over the total number of words or phrases is not relevant.

### Emergent Themes in Posts

#### Overview

The results of the thematic content analysis produced five overarching themes: (1) history of cannabis use, (2) consequences of cannabis use, (3) reasons for changing cannabis use, (4) cessation strategies, and (5) consequences of change. In addition to the descriptions provided later in the text, the intersections between themes and SAMHSA domains are presented in Table 2.

**Table 2:** Intersection of themes present in the top posts posted between 2011 and 2021 in the Reddit Cannabis Cessation Forum and the 4 domains of recovery established by the SAMHSA^a^.

Emergent themes in online cessation community posts	SAMHSA’s domains of recovery			
	Health	Community	Purpose	Home
Individual identity and cannabis use	—^b^	“when I started smoking, it was awesome - I loved going to parties and hanging out with friends. Smoking weed felt so good, so I started doing it more and more...”	“I smoked weed every day for 10 years” “Looking back, I used to feel like, ‘man, I am a relatively productive stoner’”	—
Consequences of cannabis use	“I experienced psychosis due to drug use, and attempted suicide during a period of psychosis, and yet I still smoked – I used on and off my mental health meds”	“my parents didn’t trust me anymore, because I continuously lied to them about smoking and would occasionally steal from their house when I needed cash”	“I can’t remember any of those years - those years are just a blurry fog”	“My behaviors when high were negatively impacting my entire family, and my parents were making changes to ensure a healthier environment for my siblings”
Reasons for change	“I hoped the anxiety I felt would be over when I stopped using”	“I felt embarrassed to be high around her because she didn’t use, so I started to moderate my use”	“I’ve been smoking daily for months. I feel lethargic, and like my life isn’t moving forward”	—
Cessation strategies	“I still get anxious, and when I am, I reach out to friends - we go out to eat, hang out, or play games”	“I asked my partner to help me by hiding my stash”	—	—
Consequences of change	“a year clean, and I am sleeping well”	“...over the past year since I quit, we’ve been able to rebuild a lot of trust and they are helping me get back on track with school”	“I feel sharper and able to efficiently multitask...I recently was promoted because I started to step up in my role”	—

^a^SAMHSA: Substance Abuse and Mental Health Services Administration.

^b^Not applicable.

#### History of Cannabis Use

Many posts contained reference to one’s personal history with cannabis use. Reference to personal history included disclosing the age at which the author who posted began using cannabis and the number of months or years the author used cannabis (eg, “I used daily since 16” or “I took my first tok at 13 and was a daily stoner by 14”). Social networks were also referenced regarding one’s period of active cannabis use; these included positive experiences they had with friends when using cannabis, though these times were often recalled as occurring earlier in one’s history of cannabis use.

Providing a background on the author’s personal experience with cannabis in some posts was stated as evidence against the false social perception that cannabis is not addictive (eg, “call it whatever you want, an addiction, dependence, over use, whatever – as someone who quit 97 days ago after using daily for 19 years, I can say for certain that for some people, having pot in your life means you is taking away your ability to reach your full potential”). For many authors, the reference to time was related to cessation. For some authors, it was the celebration of recent cessation (eg, “it has been five days since I last lit up!”); for others, it was to provide perspective on the experience of not having cannabis in their lives for a prolonged period (eg, “it has been 6 years since I last used the green monster...I’m proud to live a life that is free from the haze of weed”) and to sharing one’s iterative experience with cessation through chronicling experience by period (eg, “month one, no sleep; bizarre dreams if you can drift off; month two, settling in, no longer always thinking about cannabis...month six, new friendships not on the basis of smoking together”).

SAMHSA’s domains of purpose and community were reflected in this theme. Multiple authors discussed the intertwined nature of cannabis use and their daily life activities. During their active use stage, cannabis became a major feature in their daily lives, pulling their attention and resources and informing social networks. The reference to social ties with other people who actively use cannabis maps onto the SAMHSA constructs of community, while feelings of connection intersect with the construct of purpose. Nonetheless, posts suggested that the social connections formed through cannabis use may wane over time, or the cost of prolonged cannabis use on one’s health, decreased feelings of purpose, added burden on other social relationships, and risk toward home may reduce the benefits of the social connections that were built with the use of cannabis. In sum, reference to one’s history was important to many authors, reflecting times in their life that had direct implications for feelings of community connectedness as well as a sense of purpose.

#### Consequences of Cannabis Use

While there were some perceived benefits to cannabis use, particularly noted when individuals started using, authors noted many negative consequences. Authors most frequently identified consequences as an effect of prolonged, frequent cannabis use. These consequences were related to SAMHSA’s domains of health, purpose, community, and home. Health consequences included cognitive and physical effects. Fuzzy memories, a haziness, or a lack of memories of a given time when high (eg, “I can’t remember any of those years—they are just a blurry fog”) were all examples of cognitive consequences. Cognitive effects extend beyond conscious memories into sleep, in the form of not being able to remember dreams (eg, “while smoking, I never remembered my dreams”). However, in other cases, not remembering was a marker of the severity or habitual nature of cannabis use (eg, “I can’t remember the last holiday where I wasn’t high”). Cognitive effects of cannabis use also impacted the ability to maintain existing or build new social connections, and at times, it resulted in feelings of isolation and loneliness, such as an individual not having friends during their “weed-induced haze,” resulting in them feeling “antisocial” for a period of their life. Weight gain was a physical consequence noted by authors (eg, “weed made me fat—I had no ability to resist snacking”).

While some authors noted a perceived normativity of cannabis consumption during certain phases of life (eg, high school and college), many noted the level of their consumption was greater than others, with negative consequences such as worse grades and poor social interactions at work (eg, “I avoided eye contact with people I didn’t know, and when I’d speak, I’d turn red because I sounded dumb”). Some authors noted ways in which established relationships were deeply strained or even ended because of the author’s use of cannabis (eg, “my parents didn’t trust me anymore, because I continuously lied to them about smoking and would occasionally steal from their house when I needed cash”). This was particularly true for romantic relationships (eg “she got sick of me not being present, just constantly stoned—eventually, she left”). Even in cases in which established relationships were not entirely ended, some authors noted that needing a space to use cannabis limited the types of interactions in which the author and their romantic partner interacted.

Though commented on less, the SAMHSA domain of purpose was also present; some of the authors retrospectively recounted their perceptions that prolonged, frequent use of cannabis resulted in a loss of sense of self, sense of purpose, and feeling like one’s life was stuck and not moving forward (eg, “all my life was going to work then coming home and smoking; I had no inspiration, no intention—weed made me boring and stagnant”). At times, this topic included losing their sense of what they wanted for their lives, and they felt that the ongoing use of cannabis had reduced their understanding of their core values. While the SAMHSA domain of home (having a stable, safe place to live) was also less common, violations of relationship quality or trust, including stealing and lying, at times were associated with having to move out of a given living situation. Overall, posts reflected negative consequences of cannabis use related to all 4 of the SAMHSA recovery domains (health, community, purpose, and home).

#### Reasons for Change

Throughout posts, authors shared various motivators for changing their use of cannabis. Sometimes this was expressed as a broader sentiment (eg, “I know stopping would be good for my life”). At other times, specific motivations were mentioned, such as a desire to mitigate cognitive consequences. For example, the loss of memory was a motivator for one author who wanted to remember the last bit of time left with a beloved, but sick, pet; another author wanted to be more present and have clearer memories of time with their family. Other authors wanted to stop the “numbing effects” they experienced through frequent, prolonged cannabis use, even if these effects were desired in the early stages of cannabis consumption. Mental health was also a motivation for change; while some authors acknowledged the use of cannabis when “feeling depressed” or anxious, others noted a desire to change because of the negative effects on their mental health (eg, “I hoped the anxiety I felt would be over when I stopped using [cannabis]”). This included when individuals perceived that cannabis use increased mental health symptoms (eg, “weed makes my depression worse, and my ability to handle dealing with my depression harder”). However, changes in mental health symptoms on reduction or cessation of cannabis use were not always positive (eg, “I grow anxious when I don’t smoke”), perhaps reflecting withdrawal symptoms. In addition, even with the knowledge that cannabis may have negative effects on mental health, the negative effects did not always yield immediate behavior change (eg, “I experienced psychosis due to drug use, and attempted suicide during a period of psychosis, and yet I still smoked—I used on and off my mental health meds”).

The effects of cannabis on mental health symptoms often interacted with other SAMHSA domains of recovery, including community. For example, some authors observed in retrospect that while cannabis had initially made them feel less anxious in social settings, after a while, it made them more socially anxious and paranoid, resulting in withdrawal from social interactions, including staying home or skipping events. While relationships were at times established or ended because of cannabis use, in some cases, a relationship was a reason for the change (eg, “I felt embarrassed to be high around her because she didn’t use, so I started to moderate my use”). Finally, feelings of purpose were at times mentioned as a motivator for change; with less time spent on acquiring, using, and acutely recovering from cannabis use, some individuals noted they would be able to “focus on thriving in life as opposed to just existing.” This sentiment also extended to the ability to trust one’s internal perceptions and feel less paranoid. Across the various specific reasons for change, most can be described as related to the SAMHSA recovery domains of health and community.

#### Behavior Change Strategies

While the r/leaves community aims for cannabis cessation, many members recognize that reducing consumption might be a viable option for some people. However, they may not consider it the best choice for the specific author of the post. Choosing cessation as a goal over reduction or moderation at times reflected challenges in maintaining reduction or moderation while continuing to use cannabis. While some individuals in the community reported trying moderation as a step to cessation, moderation often did not help many authors cease their cannabis use, and many of the authors reported stopping “cold turkey.”

Many of the strategies for cessation were individually guided and behavioral. For some, this was resisting thoughts (eg, urges) about wanting to resume cannabis use (eg, “I’m learning how to resist thoughts and the want to smoke”); for others, it was learning to handle challenging thoughts via new behavioral and mental coping strategies (eg, “now I write down my thoughts, as it helps me process”). Individuals found helpful strategies and tips through the online subreddit community; for example, after reading posts, one author commented they, “...wrote out a list of reasons to quit and posted it on the wall [in a common area].” This list included reasons why the person quit and reasons why they would not use cannabis again in the future. Another strategy mentioned was to “stay busy” to get through the initial months of cessation and to not return to using cannabis because of boredom or uncertainty of what to do with one’s time (and particularly periods when the person frequently used cannabis, such as at night).

Coping skills were also frequently referenced, often in conjunction with one’s social relationships. For example, individuals asked for tangible support behaviors from romantic partners (“I asked my partner to help me by hiding my stash”). Friends and family also were at times attributed as forms of support (eg, “I came clean and told my friend I needed support in order to change” or “I’m still working on making changes, but now that I quit, I have the support of my family, which is helping me”). In other cases, individuals noted spending time with friends rather than giving in to triggers to use cannabis (eg, “I still get anxious, and when I am, I reach out to friends—we go out to eat, hang out, or play games”). While social relationships provided a mechanism of support, some individuals noted the need to set boundaries within existing social relationships as a way to sustain cessation (eg, “at my friend’s cook out, I avoided my friends who smoked—I was worried I couldn’t say no”). Behavioral change strategies were largely related to the SAMHSA recovery domain of health, whereby individuals were finding ways to choose behaviors that helped them achieve their goals of reduction or cessation, and several of the behaviors reflected the role of social support in behavior change, mapping onto the recovery domain of community.

#### Consequences of Change

There were positive (ie, benefits) and negative consequences of change, touching upon the SAMHSA recovery domains of health, community, and purpose. For health, sleep and dreams were often referenced both as positive and negative consequences. Reflecting on short-term effects, some individuals noted difficulties with sleep (eg, “sleeping isn’t happening”). Others found themselves having “vivid,” “wild” dreams. However, after some time, other individuals noted that they had returned to better sleep (eg, “a year clean, and I am sleeping well”), which was a benefit of change. Another outcome related to physical fitness, with some individuals noting that they spent more time working out as both a coping strategy (ie, distraction) and as a way to invest in their health and well-being (eg, “I started small, just committed to getting to the gym every day; now I can lie down at night with a body that feels good and tired from a 10 mile bike ride”). Others noted changes to eating habits, either through less snacking because of not being intoxicated or as an intentional way to invest in their behavior change and their health by choosing healthier options for meals.

Cessation was often associated by authors as having effects on their social networks. Sometimes, this was positive because of improving previously strained family relationships (eg, “My behaviors when high were negatively impacting my entire family, and my parents were making changes to ensure a healthier environment for my siblings...over the past year since I quit, we’ve been able to rebuild a lot of trust and they are helping me get back on track with school”). Other times, this meant more time to invest in existing social or romantic relationships (“I now spend more intentional time with my partner—I’m not just thinking about when I can go home and get high, but actually pay attention to what we are doing”). Sometimes, new relationships were formed (eg, “I’m dating someone new and they are really awesome and motivated to live a healthy and positive life”). There were also many references to the support that individuals received through the r/leaves subreddit; in some cases, this seemed to help support individuals who were moving away from relationships that were associated with active cannabis (eg, “I gave up my weed friends—I’m really thankful for finding support in this sub[reddit]”).

Changes in feelings of purpose because of cannabis behavior change were positive. Within work and school settings, multiple authors noted feeling more productive and having an increased ability to engage with the content or tasks associated with their role because they no longer felt the mental haze of being high (eg, “I feel sharper and able to efficiently multitask...I recently was promoted because I started to step up in my role”). Others felt a better sense of self and direction because of behavior changes (eg, “I’m focusing on defining new goals for my life, one of which I realized was going back to school!”). Other authors noted that they were able to engage in activities that they enjoyed in their life and be more present within those experiences, which added meaning and new insights. Finally, multiple authors noted the importance of helping others; sometimes, this was general advice (eg, “go help others, giving back makes life more meaningful”). Other times, the reflection on helping others was within the r/leaves subreddit community (eg, “I’m grateful for the support I’ve gotten from this community, and I want to share my story in case it helps others to stop and to feel more fulfilled”). Individuals discussed many outcomes that they attributed to reducing or ceasing cannabis use within the online forum. These outcomes align with the SAMHSA recovery domains, including making choices to promote health, building connections with family and community, and engaging in activities that provide a sense of purpose.

## Discussion

### Principal Findings

Our findings provide information to help fill 3 important gaps in knowledge on the use of a nonclinical, online cannabis cessation community. First, our findings show that the use of this cannabis recovery forum has increased in recent years, with >80 times the number of posts in 2021 as compared to 2011. This finding supports and expands upon data showing increases in engagement with online mutual help organization cannabis support groups (ie, Marijuana Anonymous) [26]. Second, we identified 5 major themes in the online community: individual history and cannabis use, consequences of cannabis use, motivators for cannabis use change, cessation strategies, and consequences of change. Within these themes, we identified intersections with all 4 of the SAMHSA domains of recovery (health, community, purpose, and home). Our findings taken together highlight the importance of online communities, both in their growing popularity and in their content supporting multidimensional definitions of recovery.

We hypothesized that increases in the community would exist, as evidenced by a higher number of users and posts over time. We found that the rate of increase was not constant but differed based on the period. Specifically, we found that the increase in annual posts steepened around 2018. This temporal trend may be explained by several factors. First, the rise in forum use may be related to the general expansion of Reddit [28]. Second, during this period, several US states legalized cannabis for recreational purposes (eg, Michigan in 2018, Illinois in 2019, and 8 additional states in 2020-2021), which coincided with increases in CUD [50], potentially in part because of the increased risks of dependence posed by higher tetrahydrocannabinol content in legalized cannabis [10,12]. However, it is important to note that not all Reddit users are from the United States and that because of the anonymous nature of community members, it is not possible to determine the demographic composition of the r/leaves community [39]. Regardless, the increased involvement in this community reflects a greater absolute number of individuals seeking support within the online space to quit or reduce cannabis consumption. The increased use supports the notion that online spaces such as r/leaves provide an accessible forum to support those seeking recovery from cannabis.

### More Than Just Change: Understanding the Context of Cannabis Use as a Critical Aspect of One’s Narrative

The most popular content of the r/leaves forum was the progression from beginning cannabis use through desired changes, strategies for change, and outcomes attributed to change. While consequences of cannabis use, reasons for change, strategies for change, and outcomes related to change may be expected given the r/leaves community mission statement of being “a support community to help stop smoking cannabis, marijuana, pot, weed, edibles, or getting high,” the importance of the narrative arc of the circumstances of when someone began using cannabis, including reasons why, and the frequency that individuals listed their duration of use emerged in many posts. For many individuals, cannabis was present for a significant amount of their lives, with authors frequently reflecting on years of use; it appears that sharing the long-standing presence of cannabis in the author’s life was important to the author and meaningful in their interaction with the online community. Our findings that narratives were common complemented and expanded other research. Narrative storytelling is common in other nonclinical substance recovery organizations, such as Alcoholics Anonymous, and has been theorized to be a mechanism through which individuals maintain their behavior change [51]. In addition, telling one’s story can help reduce forms of stigma and increase help-seeking behavior [52], while narratives with examples of successful substance use change can help inspire those who read the stories to make their own behavior changes [53]. However, unlike groups such as Alcoholics Anonymous, in which individuals may share their stories multiple times throughout their attendance, we found that almost 90% (797/965) of posts within this cannabis cessation community were written by unique authors. Future work that explores repeated engagement in telling one’s own story versus singular sharing may be important to better understand this contrast. In addition, future research could examine if individuals repeatedly share aspects of their story within comments to other posts.

### Content of Posts: Intersections With SAMHSA Domains of Recovery

There were many cases of overlap between the 5 themes found in this online nonclinical cannabis cessation community and SAMHSA’s 4 domains of recovery (health, purpose, community, and home). For example, references to health in the themes of active cannabis use experiences and history of use, motivations for change, and consequences of change included anxiety and depression. Although there is research into the potential medical uses of cannabis related to mental health [54,55], it is noted that r/leaves focuses on CUD, and the findings herein align with well-documented research from clinical settings on the bidirectional association between greater cannabis use and poorer mental health symptoms. Specifically, multiple studies document the association of greater cannabis use with greater anxiety and depression [56-58], while other researchers have found that cessation of cannabis use increases the likelihood of withdrawal symptoms, which can include acute anxiety and mood changes [14].

While physical health topics such as sleep and dietary choices were sometimes referenced within negative consequences of cannabis use, they were more often talked about within outcomes of change. Sleep was at times negatively impacted by cessation, particularly in the acute period directly after cessation, which is likely a symptom of withdrawal [54]. Specifically, multiple posts referenced a lessening of sleep disturbances over time and the return to perceived normal sleep. In contrast, dietary choices within consequences of change were often referenced as a means of identifying purpose and investing in one’s self. While research has shown that cannabis use does not always increase BMI and cannabinoids may increase metabolism and thus decrease BMI [55], identifying food choices as a mechanism of change with the motivation of investing in one’s health could be leveraged in both clinical and nonclinical settings as another pathway through which individuals might successfully achieve their intended behavior changes.

Cognitive impairment (sometimes referred to as “fog” or “haze” by authors) was also commonly mentioned in this online community and appeared across multiple themes. Previous research has described the contrasting motivations of using cannabis to both tune out and disconnect from life as a form of relaxation as well as to tune in and focus intensely on a given point of stimuli [56]. Our findings supported this, as authors in the cannabis online community referenced mental haze or fog as the aim of cannabis use to tune out stressors of life within the theme of cannabis use and history of use. However, the cognitive effects of cannabis use were perceived to have negative impacts on social relationships, health, and sense of purpose. Additional work identifying the levels or contexts of cannabis use that cause or increase mental health symptoms and contrasting them to contexts under which cannabis use may be medically relevant are important future directions and could be used to inform future public health campaigns to enhance public awareness and knowledge.

Social relationships were referenced across all 5 themes identified, highlighting their importance. Social relationships are part of SAMHSA’s domain of community, which includes “relationships and social networks that provide support, friendship, love, and hope” [15]. Though cannabis use does not always result in severe social consequences, it can negatively impact one’s social relationships and ability to engage in prosocial daily activities [22]. In posts, we found that individuals managed their social relationships during cannabis cessation, with, at times, the need to instill relational boundaries that would help to prioritize their cannabis cessation goals. Cessation also appeared to prompt rebuilding relationships that had become strained during one’s period of cannabis use, such as with parents or family members, and the opportunity to build new relationships outside the context of using cannabis. The prevalence of social relationships mentioned throughout posts and the inclusion of social relationships within the SAMHSA domain of community stresses the importance of these interpersonal ties when seeking recovery from cannabis use. Although we found a high prevalence of posts related to social relationships, other forms of community building, which have been identified in recovery from addictions to other substances [57], such as engagement with religion or community service, were less common in posts. It may be that individuals who would benefit from community building through religion or service may not use this online community or that these themes may not be as popular.

The SAMHSA recovery domain of home (a stable, safe place to live) appeared less in this online community. Though there was a slight reference to home in regard to negative consequences of use, in that cannabis use was at times stressing one’s social relationships with those that they resided with, one hypothesis may be that CUD is less commonly associated with housing insecurity [58]. Alternatively, it may be that those who experience housing insecurity because of cannabis use have less access to the online space because of barriers to access (eg, Wi-Fi or data, computers or smartphones, and electricity to charge phones), or this online space is of much lower priority given other needs. Further exploration of the impact of cannabis on housing is needed outside of this online community to better understand the risk. This research would also help to inform which populations are not served by the type of nonclinical online community examined in this study.

### Limitations

Though this study provides important findings on an emerging area of research, it also has limitations. First, we only analyzed the content of the initial post; while providing important information on the types of topics that individuals are seeking to discuss, it does not provide information on the depth and content of discussions that may have occurred in response, which could be a useful direction for future work [59]. Second, though the Pushshift API is a widely used resource [31,60,61], it has some limitations [38], including in relation to missing data (eg, missing data for the r/leaves subreddit in the year 2013) because of the nature of the archival process; however, in analyses on missingness, researchers found that missingness is not differential by subreddit [62]. In addition, despite the year of missing data, the increase of posts across time within the r/leaves subreddit is significant and thus does not undermine our findings of growth. Finally, selecting the top 100 posts based on the score within Pushshift is a limitation. This is because while the Pushshift API catalogs data at regular intervals, it is inherently an archival tool. However, posts can exist and can be viewed and commented on indefinitely [63]. By creating a static archive, any interactions that occurred between the most recent pull of the cataloging process may not be reflected in the Pushshift data set. While this can create some bias in the Pushshift data set, it is important to contextualize this limitation within the regular life cycle of a post; the first few hours of a post’s “life cycle” is when a post typically receives the majority of its views and interaction, after which the post starts to move downwards in the feed, moving to later pages on the subreddit, and thus receives far fewer views or interactions [63,64]. Thus, the catalog process has likely captured most of the activity. In addition, the use of an archived data set increases the replicability of findings, which is critical in building empirical knowledge.

### Conclusions

In summary, nonclinical online support communities may be an important resource for individuals who desire to change their cannabis use. We found that the popularity of this forum increased by >8000% from 2011 to 2021. The 5 major themes of discussion represented a narrative arc, covering the proactive engagement in cannabis use, the negative consequences of use, strategies for changing behaviors, and the positive and negative outcomes of those changes. The significant overlap between the themes present in this online community and SAMHSA’s 4 domains of recovery supports the fact that this community is engaging in meaningful discussions that overlap with aspects of clinically supported recovery, despite these interactions occurring outside of a clinical setting. Future research should examine the interactions of posts to understand forms of support, information and resources provided, and gaps in the types of content shared as well as to inform opportunities for future online interventions and referrals to online communities for support.

## Abbreviations

API: application programming interface
CUD: cannabis use disorder
SAMHSA: Substance Abuse and Mental Health Services Administration

## Data Availability

Data are not available to share due to Department of Veterans Affairs and ethical retractions; however, annotation instructions and vocabulary are available upon request..
